# Complete Genome Sequences of Two Porcine Enterotoxigenic Escherichia coli Strains

**DOI:** 10.1128/genomeA.00059-18

**Published:** 2018-02-22

**Authors:** Zhaoli Li, Ningning Song, Weijie Li, Philip R. Hardwidge, Zhigao Bu, Siguo Liu

**Affiliations:** a State Key Laboratory of Veterinary Biotechnology, Harbin Veterinary Research Institute, Chinese Academy of Agricultural Sciences (CAAS), Harbin, China; b China Institute of Veterinary Drug Control, Beijing, China; c Department of Diagnostic Medicine/Pathobiology, Kansas State University, Manhattan, Kansas, USA; d Jiangsu Co-innovation Center for Prevention and Control of Important Animal Infectious Disease and Zoonoses, Yangzhou, China

## Abstract

Enterotoxigenic Escherichia coli (ETEC) is one of the main causes of illness and death in neonatal and recently weaned pigs. Here, we sequenced the genomes of two ETEC strains that were previously used as inactivated vaccines in China.

## GENOME ANNOUNCEMENT

Enterotoxigenic Escherichia coli (ETEC) is one of the main causes of illness and death in neonatal and recently weaned pigs (1). The manifestation of ETEC infection in piglets is diarrhea, which causes heavy economic burdens to pig farmers because of the usage of drugs, the loss of pig weight, and even death of the piglets (2). ETEC strains CV839-15 and CV839-06, originally isolated from diarrheic piglets in England and the United States, were used as inactivated vaccines years ago, and to some degree, the vaccinated piglets were protected from ETEC infection in China (Harbin Veterinary Research Institute, personal communication). Since vaccine registration has become stringent in China, these strains are no longer used for vaccine production. However, the protective efficacies of the inactivated ETEC vaccines prompted us to sequence the genomes of these two strains to understand better the mechanisms of vaccine-mediated protection.

Genomic DNA was extracted from overnight cultures of E. coli strains CV839-06 and CV839-15 using a bacterial total DNA extraction kit (Qiagen, USA). Illumina MiSeq PE (300 bp) and PacBio fragment (8 to 10 kbp) libraries were constructed, and raw sequence data were obtained using the combined methods of the Illumina MiSeq system and PacBio single-molecule sequencing. Data analysis and genome assembly were performed using the bioinformatics tools BLASR, Celera Assembler 8.0, and GapCloser version 1.12 (SOAP*denovo*) (3).

The assembled genome sequences were submitted to NCBI GenBank. The genomes of strains CV839-06 and CV839-15 consisted of 4,931,011 bp and 4,917,635 bp, respectively. They encoded 4,795 and 4,812 proteins, respectively, as predicted by NCBI’s Prokaryotic Genome Annotation Pipeline (4). The genomes of CV839-06 and CV839-15 had 50.81% and 50.87% G+C contents and harbored 94 tRNA and 96 tRNA genes, respectively, and both encoded 22 copies of the rRNA operon. Strain CV839-06 harbored 2 plasmids, the sizes of which are 110,160 bp and 63,821 bp. The G+C contents of the plasmids are 49.39% and 43.86%. Strain CV839-15 harbored 3 plasmids, the sizes of which are 86,835 bp, 195,578 bp, and 62,669 bp. The G+C contents of the plasmids are 51.61%, 48.86%, and 51.07%.

### Accession number(s).

The genome sequences of strains CV839-06 and CV839-15 were deposited in GenBank. The accession numbers for CV839-06 and its two plasmids are CP025753, CP025752, and CP025751. The accession numbers for CV839-15 and its three plasmids are CP024978, CP024975, CP024976, and CP024977. The versions described in this study are the first versions.
